# Direct Numerical Simulation of Seawater Desalination Based on Ion Concentration Polarization

**DOI:** 10.3390/mi10090562

**Published:** 2019-08-25

**Authors:** Jie Li, Dilin Chen, Jian Ye, Lai Zhang, Teng Zhou, Yi Zhou

**Affiliations:** 1 School of Energy and Power Engineering, Wuhan University of Technology, Wuhan 430070, China; 2 Mechanical and Electrical Engineering College, Hainan University, Haikou 570228, China

**Keywords:** cross-membrane voltage, ion concentration polarization, desalination effect, pump effect, eddy current

## Abstract

The problem of water shortage needs to be solved urgently. The membrane-embedded microchannel structure based on the ion concentration polarization (ICP) desalination effect is a potential portable desalination device with low energy consumption and high efficiency. The electroosmotic flow in the microchannel of the cation exchange membrane and the desalination effect of the system are numerically analyzed. The results show that when the horizontal electric field intensity is 2 kV/m and the transmembrane voltage is 400 mV, the desalting efficiency reaches 97.3%. When the electric field strength increases to 20 kV/m, the desalination efficiency is reduced by 2%. In terms of fluid motion, under the action of the transmembrane voltage, two reverse eddy currents are formed on the surface of the membrane due to the opposite electric field and pressure difference on both sides of the membrane, forming a pumping effect. The electromotive force in the channel exhibits significant pressure-flow characteristics with a slip boundary at a speed approximately six times that of a non-membrane microchannel.

## 1. Introduction

In 1809, Reuss [1] first discovered the electroosmotic flow (EOF) phenomenon in an experiment. In the late 19th century, Helmholt [2] first introduced the concept of the electrical double layer (EDL), which links the electric field, fluid flow, and ion concentration to describe the formation mechanism of the EOF in detail. When the ion concentration in the Debye layer is constant, the fluid velocity of the EOF is linearly related to the applied horizontal electric field. When the applied electric field destroys the equilibrium condition inside the Debye layer, the ion concentration in the layer changes to cause an uneven zeta potential, causing a nonlinear electroosmotic slip near the interface of the micro-nano channel [3]. These complex unbalanced electrokinetic phenomena are important in the field of biomolecules or charged particle separation and enrichment [4,5,6]. In particular, the development of new micro-nanofluid systems capable of changing the electrolyte concentration in the EDL has once again caused researchers’ scientific interest in unbalanced EOF.

When an ion exchange membrane (IEM) can only absorb or pass a specific polar ion, these polar ions (such as cations) are subjected to a tangential electric field to reach the nanochannel (or membrane) and be absorbed. Due to the electric field force and the electrostatic repulsion of the ions themselves, the ions of the opposite polarity are far away from the nanochannel, resulting in a lower concentration of the ion depletion region, which greatly reduces the conductivity. On the other side of the nanochannel, a very high concentration of ions is formed. Therefore, ions diffuse from a high concentration region to a low concentration region near nanochannels, forming an ion concentration polarization (ICP) effect [7,8]. When the vertical electric field is strong, an extended space charge layer (ESC) is produced between the solid surface and the diffusion layer [9,10]. Under the action of the horizontal electric field, the net charge in the ESC will drive the liquid to flow along the wall, and this flow is similar to the electroosmosis of the first kind (EOF1), formed by the action of a horizontal electric field on the double layer. Therefore, it is called the second kind (EOF2) [11]. However, for the charge of these two electroosmotic flows, the charge of the ESC is at least 10 times that in the electric double layer [12,13]. In recent years, researchers have developed EOF2 on ion exchange membranes or electrode surfaces and developed unstable nonlinear eddy currents in theories [14,15], experiments [16,17], and numerical simulations [18,19,20,21,22,23]. The phenomena of ICP and the formation mechanism of EOF2 are discussed in detail [24]. EOF2 has also been successfully applied to fluid or particle drives. Mishchuk et al. [25] placed ion exchange microspheres in microchannels and successfully designed the second type of the electroosmotic micropump, which greatly increased the flow rate. Kivanc et al. [26] realized the second kind of the electroosmotic micropump using a porous silicon skeleton structure as a substrate and analyzed the influence of the substrate area and thickness on the flow rate. At the same time, using ICP and EOF2 phenomena, Han’s research team [27] used the nanochannels in microchannels to achieve efficient desalination of seawater. Several studies involving space charge models and related applications in ion-exchanged porous membranes have also attracted much attention. Sherwood et al. [28] analyzed a theoretical model of electroosmotic flow considering end effects. Yang et al. [29] developed a multi-component space charge transport model for an IEM using cylindrical pores of variable radius/charge density. Szymczyk et al. [30] also studied the pressure-driven ion transport of nanochannels with an inhomogenrous charge distribution. Moya [31] analyzed the electrochemical impedance of an IEM in two counterion ternary electrolyte solutions. Ahualli et al. [32] studied the equilibrium kinetics of the energy production of ion exchange membranes and battery capacitors. Deng et al. [33] experimentally confirmed that impact electrodialysis as a new method for water desalination and that other electrochemical separation is feasible.

At present, the research on the embedded microchannel system is mainly based on the experimental observation of the tracer particles. However, direct experimental monitoring of critical parameters (such as ion concentration) is still unrealistic, which hampers an accurate understanding of the microchannel system, and the accurate description must rely on numerical simulation. However, the impact that transmembrane voltage and horizontal voltage exert on desalination characteristics and pump efficiency has also not been studied. Based on the ideal ion exchange hypothesis (that is, only one ion is allowed to pass through the membrane surface), this study numerically simulated the dynamic characteristics of the micro-channel electric system embedded with a cation exchange membrane (CEM), and the influence of the horizontal electric field on the desalination effect and pump effect are analyzed. Below, Section 2 gives the system description, governing equations, and boundary conditions. The numerical results are given in Section 3.

## 2. Methods

### 2.1. System Setup

**Figure 1:**
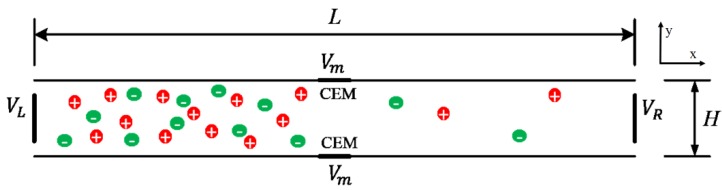
Schematic diagram of the microchannel model embedded with an ion exchange membrane (IEM).

Figure 1 shows a schematic of a two-dimensional (2D) model, which is a microchannel embedded with an IEM in the middle of both top and bottom walls. The left boundary was connected to the reservoir, which was filled with NaCl solution. The microchannel walls were negatively charged and the surface charge density was set as $\sigma_{-}$, so the channel wall would adsorb cations and induce an EDL, resulting in more cations than anions in the channel. An externally axial electric field was imposed, inducing an EOF because of the Coulomb force and fluid drag. If the potential ($V_{L}$) at the left boundary is higher than that at the right boundary ($V_{R}$), the EOF flows from left to right. Therefore, the left and right boundaries were set as the inlet and outlet of the fluid, respectively. The lengths $L_{m}$ of the IEM ($L_{m}$ << *L*, the thickness was neglected) were assumed to permit passage of Na^+^ only, and the surface potential of the membrane was set as $V_{m}$. For convenience of analysis, cross-membrane voltage $V_{cm}=\left(V_{L}+V_{R}\right)/2-V_{m}$ was defined, representing the difference between the potential at the middle of the channel without the membrane and the potential actually applied on the membrane. Larger $V_{cm}$ generated a more obvious ICP phenomenon.

### 2.2. Governing Equations and Boundary Conditions

In this study, the symmetric electrolyte NaCl solution was chosen. The governing equations of the EOF multi-physics coupling field included the Poisson–Nernst–Planck (PNP) equation and the improved Stokes equation. According to electrodynamics, in the diffusion layer the potential generated by the net charge satisfied the classical electrostatic Poisson equation:

(1) $$-\varepsilon_{0}\varepsilon_{f}\nabla^{2}\phi =\rho_{e}=F\overset{n}{\underset{i=1}{\sum }}z_{i}c_{i}$$

where $\varepsilon_{0}$ and $\varepsilon_{f}$ are the absolute permittivity constant of the vacuum and the relative dielectric constant of the fluid, respectively; ϕ is the electric potential within the solution; *F* is the Faraday constant; $c_{i}$ and $z_{i}$ are the ionic concentration and the valence of ions, respectively; and n is the number of ion species. $\sum_{i=1}^{n}Fz_{i}c_{i}$ is the net charge density $\rho_{e}$, which is the sum of all ions in the fluid.

According to ion transfer theory, ions in solution are affected by an electric field, flow field, and concentration field to form ion migration. Ion flux $N_{i}$ includes the ion convective flux, diffusion flux, and electromigration flux.

(2) $$N_{i}=uc_{i}-D_{i}\nabla c_{i}-z_{i}\frac{D_{i}}{RT}Fc_{i}\nabla \phi$$

where u is the fluid velocity, $D_{i}$ is the diffusivity of the *i*th ionic species, and *R* and *T* are the general gas constant and the absolute temperature of the solution, respectively. In the steady state and lack of fluid flow, no chemical reaction occurs in the solution and the ion flux obeys the simplified Nernst–Planck (NP) equation:

(3) $$\nabla \cdot N_{i}=\nabla \cdot (-D_{i}\nabla c_{i}-z_{i}\frac{D_{i}}{RT}Fc_{i}\nabla \phi )=0$$

The electrical force under an external horizontal electric field

(4) F=−∇ϕ

Therefore, according to the principle of conservation of momentum, the movement of fluid is governed by the improved Navier–Stokes (N–S) equation and continuous equation.

(5) $$-\nabla p+\mu \nabla^{2}u\;+F\;=0$$

(6) ∇⋅u=0

where ρ is the fluid density; *p* is the pressure; and μ is the fluid dynamic viscosity. The inertial term in the N–S equation is ignored due to a small Reynolds number.

Select the microchannel width *H* as the feature length, μ*U*_0_/*H* as the characteristic pressure, *U*_0_ =$\varepsilon_{0}\varepsilon_{f}$*R*^2^*T*^2^/(μ*HF*^2^) as the characteristic velocity, the bulk concentration $C_{0}$ as the characteristic concentration of the ion, and *RT/F* as the characteristic potential, and define $\kappa^{-1}=\lambda_{D}=\sqrt{\varepsilon_{0}\varepsilon_{f}RT/\sum_{i=1}^{2}F^{2}z_{i}^{2}C_{i0}}$ as the Debye length; the surface charge density σ corresponding to $\varepsilon_{0}\varepsilon_{f}$*RT*/(*FH*) is dimensionless, the diffusion coefficient is dimensionless according to $\varepsilon_{0}\varepsilon_{f}$*R*^2^*T*^2^/(μ*F*^2^), and they are used to control the governing equation of the EOF coupling field without simplification. It should be noted that notations with an asterisk are used for the dimensionless parameters.

(7) $$-\nabla^{*2}\phi^{*}=\frac{{\left(kH\right)}^{2}}{2}\left(c_{1}^{*}z_{1}+c_{2}^{*}z_{2}\right)$$

(8) $$\nabla^{*}\cdot \left(-D_{i}^{*}\nabla^{*}c_{i}^{*}-z_{i}D_{i}^{*}c_{i}^{*}\nabla^{*}\phi^{*}+u^{*}c_{i}^{*}\right)=0,i=1,2$$

(9) $$-\nabla^{*}p^{*}+\nabla^{*2}u^{*}-\frac{{\left(kH\right)}^{2}}{2}\left(c_{1}^{*}z_{1}+c_{2}^{*}z_{2}\right)\nabla^{*}\phi^{*}=0$$

(10) $$\nabla^{*}\cdot u^{*}=0$$

The boundary conditions are given as the following.

On the CEM surface:

(11) $$u^{*}=0,n\cdot \nabla^{*}p^{*}=0,\Phi^{*}=V_{m}\cdot \frac{F}{RT},c_{1}^{*}=2,n\cdot \nabla^{*}c_{2}^{*}=-z_{2}c_{2}^{*}\cdot n\cdot \nabla^{*}\Phi^{*}$$

On the microchannel wall (non-membrane area):

(12) $$u^{*}=0,n\cdot \nabla^{*}p^{*}=0,n\cdot \nabla^{*}\Phi^{*}=\sigma_{-}\cdot \frac{HF}{RT},n\cdot \nabla^{*}c_{i}^{*}=-z_{i}c_{i}^{*}\cdot n\cdot \nabla^{*}\Phi^{*}$$

At the inlet of the microchannel:

(13) $$n\cdot \nabla^{*}u^{*}=0,p^{*}=0,\Phi^{*}=V_{L}\cdot \frac{F}{RT},c_{i}^{*}=1$$

At the outlet of the microchannel:

(14) $$n\cdot \nabla^{*}u^{*}=0,p^{*}=0,\Phi^{*}=0,n\cdot \nabla^{*}c_{i}^{*}=0$$

### 2.3. Numerical Method and Code Validation

To ensure the accuracy and feasibility of the simulation results, we first compared the numerical prediction with the results of White et al. [34]. In the current simulation, the geometry of the non-membrane channel was set as length *L* = 1000 nm and height *H* = 100 nm. The surface charge density of microchannel walls was set as $\sigma_{-}$ = −0.001 C/m^2^. The channel was filled with NaCl solution ($z_{1}$ = 1; $z_{2}$ = −1), the initial concentration was set as *C*_0_ = 10 mM, and the externally imposed axial electric field was set at 50 KV/m. Other parameters used in this study were set as μ = 0.001 N·s/m^2^, $D_{1}$ = 1.333 × 10^−9^ m^2^/s, $D_{2}$ = 2.032 × 10^−9^ m^2^/s, *F* = 9.649 × 10^4^ C/mol, *R* = 8.31 J/(mol·K), *T* = 300 K, $\varepsilon_{f}$ = 80, $\varepsilon_{0}$ = 8.854 × 10^−12^ F/m. The pressure and normal viscous stress at both ends of the channel were 0, and the wall of the channel was the no-slip boundary condition.

In this calculation, COMSOL v5.3a (COMSOL Inc., Stockholm, Sweden) was used to solve the governing equations with specified boundary conditions. Considering the geometric and physical settings, symmetric boundary conditions were used at the upper boundary of the computing domain, so only the lower half of the channel was modeled. The inaccuracy of the boundary flow field was due to the difference between the zeta potential of the channel wall and the boundary condition of the fixed potential at the junction of the inlet and outlet with the wall. Therefore, in order to reduce the sharp change of the potential at this position, no charge was applied on the two walls 1 μm away from the entrance and outlet, respectively. The calculation domain was divided into quadrilateral meshes, and a finer mesh was adopted near the charged wall, membrane surface, channel inlet, and outlet boundary, and the total number of grids was 38,500. For steady-state analysis, a multi-frontal massively parallel sparse direct solver (MUMPS) was used to solve the fully coupled PNP-NS equation.

**Figure 2:**
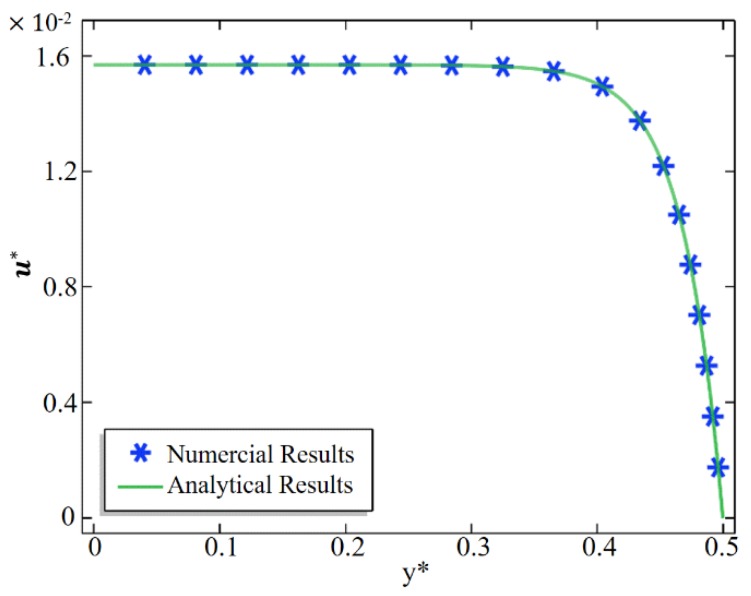
Comparison between the numerical (symbol) and analytical (solid line) results of the axial electroosmotic flow (EOF) velocity in a microchannel.

In order to verify the accuracy of the simulation results, we compared the calculated fluid velocity in the microchannel with the analytical solution of White et al. [34]. As shown in Figure 2, it is obvious that the simulation results are in good agreement with the analytical solutions. The abscissa was dimensionless $y^{*}=y/\lambda_{D}$ and the vertical axis was dimensionless $u^{*}=u/$*U*_0_.

## 3. Results and Discussion

### 3.1. Desalting Effect

Referring to Figure 1, a microchannel of the length *L* = 100 μm and height *H* = 10 μm, embedded with membranes of length $L_{m}$ = 2 μm was considered. The initial concentration of NaCl solution was 1 mM in the channel. In order to study the effect of the applied horizontal electric field strength on the ion transport and fluid flow in the channel, the inlet potential $V_{L}$ was set as a variable parameter. Due to the strong nonlinearity of the system and the inconsistency of the initial conditions of the concentration and potential near the channel or membrane surface, the inlet potential needs to be slowly increased, with a range of 0.1 V–2 V, which can be assisted by the auxiliary parameter scanning in the solver.

**Figure 3:**
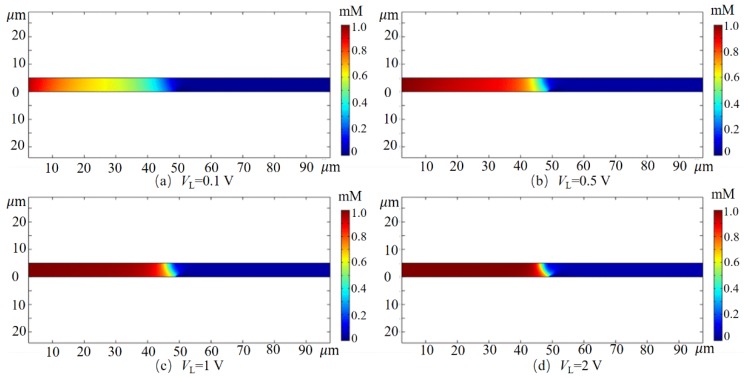
The concentration distribution of ${\mathrm{Cl}}^{-}$ in the channel at different inlet potentials at $V_{cm}$ = 400 mV.

As shown in Figure 3, the concentration distribution of ${\mathrm{Cl}}^{-}$ in the channel is given when the cross-membrane voltage, $V_{cm}$ = 400 mV, and the potential at the inlet of the channel is 0.1 V, 0.5 V, 1 V and 2 V, respectively. It is observed that the inlet potential increases and the concentration of ${\mathrm{Cl}}^{-}$ at the upstream of the channel correspondingly increases. When the inlet potential is low (such as $V_{L}$ = 0.1 V), the average electric field strength applied to the channel level is 1 kV/m, and the velocity of the formed EOF1 is small, so the fluid drives ${\mathrm{Cl}}^{-}$ to the right. The drag of the motion is small, and the strong electric field formed on the surface of the film due to the ICP phenomenon acts on the electric field force of ${\mathrm{Cl}}^{-}$ (to the left) to be greater than the drag force for the reverse motion, so ${\mathrm{Cl}}^{-}$ is concentrated upstream of the channel. However, when the inlet potential is high (such as $V_{L}$ = 1 V), the average electric field of the channel is significantly enhanced, the fluid drag is greater than the electric field force, and the ${\mathrm{Cl}}^{-}$ aggregation zone migrates to the right. When $V_{L}$ = 2 V, the ${\mathrm{Cl}}^{-}$ aggregation zone is very near the membrane surface area.

When the transmembrane voltage is $V_{cm}$ = 0 (corresponding to no membrane in the channel), the cation is adsorbed on the channel wall surface and the membrane surface to form an EDL. Under the action of a horizontal electric field, a typical EOF1 appears in the channel. The concentration of cations in the EDL is greater than the concentration of the anions, while the concentration of ions outside the EDL is substantially constant. Applying a transmembrane voltage across the membrane encourages more cations to be expelled out of the system quickly through the CEM, while the anion remains in the upstream of the channel due to the repulsion of the strong electric field in front of the membrane, forming an ion depletion zone near the membrane. As the solution flows, the ion depletion zone expands downstream, eventually forming a very low concentration of fresh water zone downstream of the channel, and then fresh water flows out of the outlet to achieve desalination.

**Figure 4:**
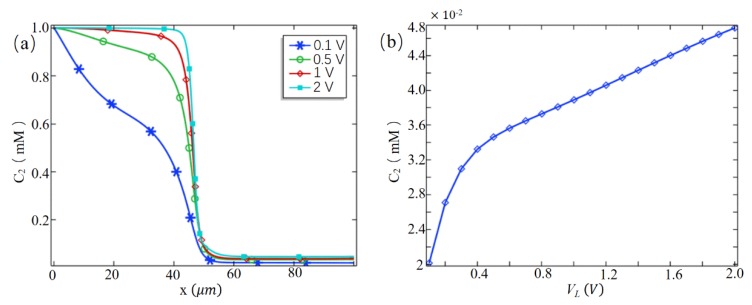
(**a**) The concentration curve of ${\mathrm{Cl}}^{-}$ along the channel symmetry line at different inlet potentials; (**b**) at the outlet, the average concentration of ${\mathrm{Cl}}^{-}$ with the inlet potential.

In order to better calculate the desalination efficiency of the system, Figure 4a shows the variation curve of the ${\mathrm{Cl}}^{-}$ concentration along the channel symmetry line when the inlet potential is 0.1 V, 0.5 V, 1 V, and 2 V, respectively. As can be seen, with the increase of entrance potential, the ${\mathrm{Cl}}^{-}$ concentration downstream of the channel does not change much. In order to obtain a specific data change, as shown in Figure 4b, when $V_{L}$ = 0.1 V, the ${\mathrm{Cl}}^{-}$ concentration is the smallest (*C*_2_ = 0.02 mM), and the desalting efficiency is as high as 98%. As the inlet potential increases, the ${\mathrm{Cl}}^{-}$ concentration gradually increases. When $V_{L}$ = 2 V, the ${\mathrm{Cl}}^{-}$ concentration is increased to 0.047 mM, and the desalination efficiency is reduced by 2.7 percent.

### 3.2. Pump Effect

**Figure 5:**
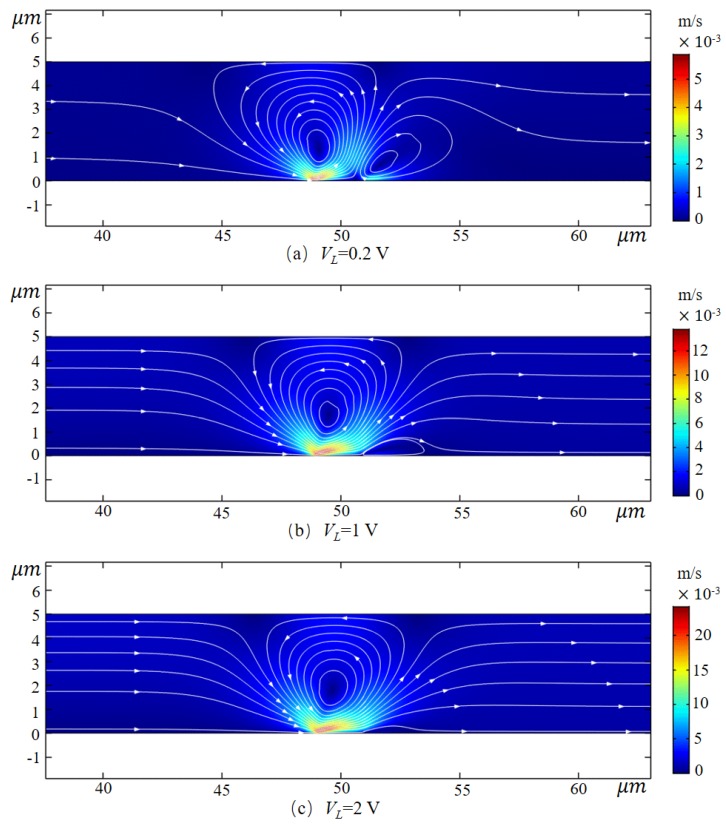
Velocity diagram of the velocity near the endometrial position (49 ≤ x ≤ 51 μm) at different inlet potentials at $V_{cm}$ = 400 mV.

Figure 5 shows the velocity streamlines around the channel intima position (49 ≤ *x* ≤ 51 μm) at $V_{cm}$ = 400 mV with potentials at the channel inlet of 0.2 V, 1 V and 2 V, respectively. When $V_{L}$ = 0.2 V, the maximum velocity of the fluid in the channel is 5.88 × 10^−3^ m/s, which is 294 times the EOF1 velocity in the non-membrane microchannel. Nonlinear vortices near the surface of the IEM or at the junction of the microchannel and the nanochannel are unique phenomena of such systems. Kim et al. [35] observed this in the experiment.

**Figure 6:**
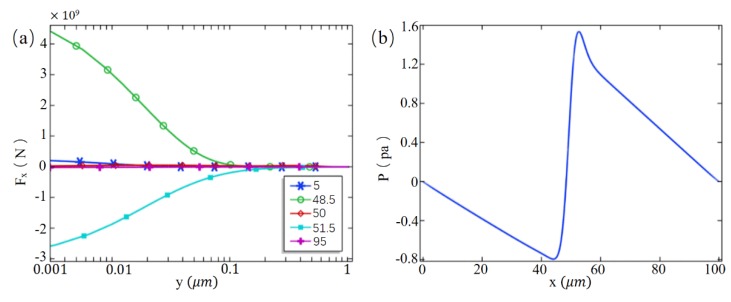
(**a**) Horizontal electric field force in the y direction at different coordinate values of the x-axis ($V_{cm}$ = 400 mV); (**b**) The curve of the pressure along the line of symmetry of the channel.

It is worth noting that there are two distinct nonlinear eddy currents near the ion exchange membrane. In order to study the formation mechanism of the eddy current, the variation law of electric field force and pressure difference in the channel was analyzed. Figure 6a shows the horizontal electric field force of the fluid at different positions on the wall along the y-axis when $V_{cm}$ = 400 mV. The fluid at the membrane side and wall junction (*x* = 48.5 μm, *x* = 51.5 μm) is subjected to the maximum electric field force, and the range of action is much larger than that of the channel inlet and outlet (*x* = 5 μm, *x* = 95 μm) and the membrane center (*x* = 50 μm). The direction of the electric field force on the upstream side (*x* = 48.5 μm) is positive, while that on the downstream side (*x* = 51.5 μm) is the opposite. The electric field force gradually decreases as it goes further from the wall surface and decreases to the same size as the channel inlet and outlet area at 10 λd from the wall surface. Under the combined action of forces, the pressure in the center of the channel has changed greatly. As shown in Figure 6b, at $V_{cm}$ = 400 mV the pressure tends to change along the direction of the symmetry line of the microchannel. Due to the electrodynamic force, the pressure on the upstream side of the membrane decreased significantly, while on the downstream side of the membrane, the pressure difference on both sides of the membrane reached about 2.4 Pa, which produced a certain pumping effect.

Therefore, the fluid on the upstream side of the membrane moves rapidly to the right due to the electric field force, while the fluid on the downstream side of the membrane moves to the left, while the large pressure difference on both sides of the membrane forces the fluid upstream in the central region of the membrane (*x* = 50 μm). The direction flows back, thereby creating a vortex that rotates in a counterclockwise direction. Since the reverse electric field near the downstream side wall surface if the membrane is strong, a small eddy current rotating clockwise is formed there. As the horizontal electric field strength increases, the small eddy current gradually decreases or even disappears.

**Figure 7:**
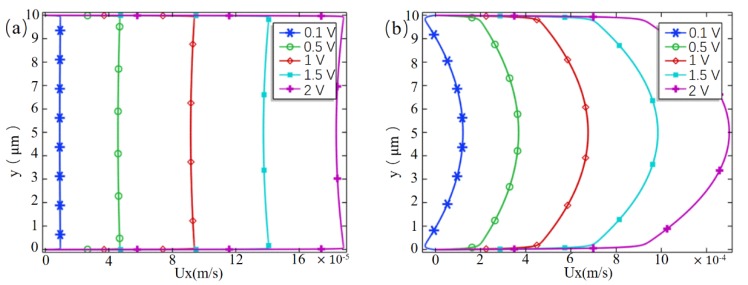
(**a**) Tangential velocity distribution of the cross section of the channel at *x* = 90 μm when $V_{cm}$ = 0 mV; (**b**) tangential velocity distribution of the cross section of the channel at *x* = 90 μm when $V_{cm}$ = 400 mV.

In order to investigate the flow characteristics of fluids in the embedded microchannel at the different horizontal electric field, Figure 7 shows the tangential velocity distribution of the channel near the outlet (*x* = 90 μm) at $V_{cm}$ = 0 mV and $V_{cm}$ = 400 mV, respectively. When the cross-membrane voltage is 0, a plunger-like distribution of typical EOF1 is formed downstream of the channel, and when $V_{cm}$ = 400 mV, it shows a significant parabolic distribution with the slip boundary, that is, the downstream of the channel is dominated by the pressure flow. When $V_{L}$ = 0.1 V, the average velocity of the fluid at the outlet is about 6.38 × 10^−5^ m/s, which is about six times that of the EOF1 in the non-membrane microchannel with the same parameters (about 1 × 10^−5^ m/s). Therefore, the embedded microchannel has a stronger fluid drive capability than the conventional microchannel. When $V_{L}$ = 2 V, the difference of the average velocity is almost constant, which means that the increase of the applied horizontal electric field has little effect on the pump effect.

## 4. Conclusions

The fully coupled PNP-NS equation is solved numerically. A microchannel simulation model with a cation exchange membrane embedded on the wall was constructed, and the response characteristics of the embedded microfluidic system under different horizontal electric fields were analyzed. In summary, increasing the horizontal electric field will reduce the desalination effect of the system. At the horizontal electric and transmembrane voltages, the fluid flow in the channel exhibits the dual characteristics of electroosmotic flow and pressure flow. The flow rate is about six times higher than the conventional electroosmotic flow, and the embedded microchannels exhibit a significant pumping effect. The mechanism of the nonlinear eddy current on the surface of the ion exchange membrane is expounded, which provides a theoretical basis for the design of a new electroosmotic pump and desalination device.
